# Hospital Meetings

**Published:** 1903-07-18

**Authors:** 


### HOSPITAL MEETINGS.

### ROYAL ORTHOPÆDIC HOSPITAL.

PROPOSED AMALGAMATION WITH THE NATIONAL.

A SPECIAL Court of the Governors of the Royal Orthopædic Hospital, Hanover Square, was held on Wednesday, 8th instant, to consider the scheme for amalgamation with the National Orthopædic Hospital, explained in the following letter which was issued to the Governors:—

*June 26th*, 1903.

LADIES AND GENTLEMEN,—In the Report presented at the Annual Court held on February 26th last, we informed you that, having disposed of our hospital site in Hanover Square, we had acquired the lease of a site for a new hospital in Coram Street, Bloomsbury, and commenced the construction of the first portion of the building.

Since then we have been approached by King Edward's Hospital Fund for London with a suggestion that we should confer with the representatives of the Fund as to the expediency of amalgamating the Royal Orthopædic, the National Orthopædic, and the City Orthopædic Hospitals. After a preliminary conference with the representatives of the two other hospitals, we informed the representatives of the King's Fund that we had already acquired a site for a new hospital of our own, and commenced building on it, and that to abandon that site might involve our institution in considerable loss.

This point was duly considered, with the result that on March 18th the Distribution Committee of the Fund passed and communicated to us the following resolution:—

King Edward's Hospital Fund for London will, as an initial step to the amalgamation of the three Orthopædic Hospitals, indemnify the Royal Orthopædic Hospital to an amount not exceeding £3,000 on any loss they may suffer in respect of the disposal of their site in Coram Street.

As a result of a further communication, we received from the honorary secretary of King Edward's Hospital Fund, on April 8th, a letter, of which the following is a copy:—

"H. H. MARKS, Esq.,
"Chairman, Royal Orthopædic Hospital,
"297 Oxford Street, W.

"8th April, 1903.

"DEAR SIR,—Will you kindly bring the following resolution to the notice of the Committee of your Hospital? It has been sanctioned by his Royal Highness the Prince of Wales, President of this Fund, and approved by the Executive Committee of this Fund—

That the Distribution Committee recommend that a grant of £10,000 be made towards the cost of the amalgamation of the three Orthopædic Hospitals and of the necessary buildings, and an annual grant of £2,000 per annum for three years commencing from a date to be agreed upon. Should the City Orthopædic Hospital not join in the amalgamation the above sums to be reduced by one-fourth, respectively.

I shall be glad to hear further from you on the matter.

"Yours faithfully,
(Signed) "SAVILE CROSSLEY,
"Honorary Secretary."

In view of this assurance we felt justified in continuing the negotiations with a fair prospect of a satisfactory issue, and we instructed our architect to suspend the building works in Coram Street. Your committee being unanimously in favour of an amalgamation upon certain lines, a series of conferences and a long correspondence have been carried on for the past three months with the representatives of the National and the City Orthopædic Hospitals and the secretaries of the King's Fund, with the following results:—

The City Orthopædic has declined for the present to join in the amalgamation.

The representatives of the Royal and the National have agreed, subject to the approval of their respective governors, to amalgamate under one management subject to the following conditions:—

1. That their joint institutions shall consist of two estabishments, viz, one central hospital, and a convalescent home or outpost hospital in a suburb of London;

2. That the site of the National Orthopædic Hospital in Great Portland Street, together with the extensions on an adjacent site, generously granted by Lord Howard de Walden, at a nominal ground rent, shall be the site of the new hospital;

3. That all beds in the hospital shall be free;

4. That the first committee of the new hospital shall be the combined committees of the two existing hospitals, each hospital contributing an equal number of members;

5. That the medical staff of the new hospital shall be the combined medical staffs of the two existing hospital; and

6. That, subject to the authority of the Courts, where necessary, the funds of the two institutions should be pooled.

We may add that the representatives of the National have agreed, in the event of necessity, to afford temporary facilities for the reception of in- and out-patients of the Royal, pending the completion of the new hospital.

Your committee share the opinion of the King's Fund that greater efficiency in the treatment of patients and greater economy in the cost of administration may be secured by such a scheme of amalgamation as is now projected; and, having regard to the opinions of hospital experts upon this matter, and the generous offers that have been made by the King's Fund to give effect to their views, your committee strongly recommend the Governors to give their sanction to the scheme of amalgamation.

A Special Court will be held at the hospital building in Hanover Square, on Wednesday, July 8th, at 4 o'clock in the afternoon, for the purpose of considering the matter, and it is hoped that it may be convenient for you to be present on that occasion.

We are, etc.,
Denbigh, *President.*
H. H. Marks, *Chairman.*
Ernest Flower, *Deputy Chairman.*

Tate S. Mansford, *Secretary.*

A circular letter was also sent out signed by several Governors as a protest against the scheme. On attending to report the proceedings our representative was informed that the meeting was to be private, and that the Press would not be admitted. We are informed, however, that an alternative scheme was submitted for rebuilding the hospital on its present site in Hanover Square, but was ruled out of order, that site having been already sold. The amalgamation scheme was then carried.

